# Classification of Sleeping Position Using Enhanced Stacking Ensemble Learning

**DOI:** 10.3390/e26100817

**Published:** 2024-09-25

**Authors:** Xi Xu, Qihui Mo, Zhibing Wang, Yonghan Zhao, Changyun Li

**Affiliations:** 1 School of Computer Science, Hunan University of Technology, Zhuzhou 412007, China; 18878251016@163.com (X.X.); wzb@hut.edu.cn (Z.W.); m22077500018@stu.hut.edu.cn (Y.Z.); 2 Hunan Provincial Key Laboratory of Intelligent Perception and Processing Technology, Hunan University of Technology, Zhuzhou 412007, China

**Keywords:** sleep posture recognition, enhanced stacking model, Bayesian optimization, entropy weight method

## Abstract

Sleep position recognition plays a crucial role in enhancing individual sleep quality and addressing sleep-related disorders. However, the conventional non-invasive technology for recognizing sleep positions tends to be limited in its widespread application due to high production and computing costs. To address this issue, an enhanced stacking model is proposed based on a specific air bag mattress. Firstly, the hyperparameters of the candidate base model are optimized using the Bayesian optimization algorithm. Subsequently, the entropy weight method is employed to select extreme gradient boosting (XGBoost), support vector machine (SVM), and deep neural decision tree (DNDT) as the first layer of the enhanced stacking model, with logistic regression serving as the meta-learner in the second layer. Comparative analysis with existing machine learning techniques demonstrates that the proposed enhanced stacking model achieves higher classification accuracy and applicability.

## 1. Introduction

Sleep is considered one of the most essential human activities, playing a critical role in bodily recovery and maintenance [1]. On average, individuals spend approximately eight hours each day sleeping, which amounts to a third of their lifetime [2]. Recent research into the relationship between sleep and health emphasizes a significant correlation, highlighting that good sleep quality is crucial to overall well-being as it enhances work efficiency and strengthens the immune system [3]. Conversely, suboptimal sleep patterns are linked to a variety of health issues [4,5,6,7,8]. Specifically, prolonged immobility during sleep can result in pressure ulcers for those confined to bed, causing damage to the skin and underlying tissues [9]. Meanwhile, for individuals with sleep apnea, side sleeping is recommended; however, studies [10,11] indicate that this position may have adverse effects on those with impaired baseline cardiac function. As a result, the identification of appropriate sleep postures has become increasingly important in managing such chronic conditions.

In the field of sleep posture recognition research, pressure sensing in key body areas is essential. Biomechanically, the waist, back, and hips serve as the primary support points for the body [12]. The location and distribution of pressure in these areas play a crucial role in determining spinal curvature and the overall stability of the sleeping position [13,14]. Studies on sleep physiology [15,16,17,18] have demonstrated that by detecting shoulder and hip pressure as well as spinal load, we can determine whether individuals maintain a posture during sleep that minimizes physical stress and supports natural spinal curvature. This understanding is important for preventing sleep-related diseases and provides a theoretical and practical basis for using shoulder–hip features and spinal features as key identifiers of sleeping positions. The main contributions of this paper are as follows:

1. Utilizing specific air bag mattress pressure data, a refined stacking learning model has been developed to accurately classify common sleeping positions. This model incorporates the predictive capabilities of multiple base learners, significantly enhancing the accuracy and robustness of classification through optimized fusion strategies. Furthermore, it introduces an innovative methodology in the field of sleeping position recognition.
2. The statistical features are extracted from the dataset, and the derived attributes are combined with the original data to form the final model input. Furthermore, the parameters of models such as SVM, XGBoost, and DNDT are further refined through Bayesian optimization in order to enhance model performance.
3. We utilized the entropy weighting method to objectively allocate weights to the five key performance indicators of each model and combined them to compute the entropy-weighted composite score. Based on this score, we selected the top three performing models as the base learners for the initial stacking model, which laid the groundwork for developing the final sleep posture recognition model.

## 2. Related Work

In recent years, the field of sleep recognition has primarily focused on three main areas: detection methods using wearable technology [19], detection techniques based on visual cues [20], and methods that utilize pressure sensors [21]. All of these methods involve classification by collecting data from devices and then performing feature engineering to extract effective features. Wearable device-based detection methods utilize accelerometers to obtain body movement data [22], but prolonged wear may affect user comfort, especially in sleep monitoring. The intrusive nature of wearable devices may interfere with normal sleep patterns, and the need for regular recharging limits the prolonged use of the devices. Vision-based detection methods [23,24] utilize a camera to acquire images of sleeping postures for classification, which, although non-invasive, are sensitive to lighting conditions and have privacy issues, especially in public or semi-public environments such as hospitals.

In contrast, the pressure sensor-based method is less intrusive to the user and offers higher recognition accuracy. For example, a study utilized an air pressure mattress combined with a data acquisition system [21] consisting of components such as the STM32F103RCT6 and STC15F2K60S2 microcontrollers for information gathering. This setup incorporated a 121-sensor array (arranged in an 11 × 24 grid) to collect pressure data from various body parts. Subsequently, Spearman’s rank correlation feature extraction was applied along with horizontal and vertical segmentation to develop an AdaBoost-SVM ensemble classifier, resulting in the creation of the SPR-DE model. Experimental results demonstrated the model’s high accuracy and robustness in posture identification, achieving a 99.9% accuracy rate and an F1 score of 0.998 by classifying sleeping postures through pressure data collection. Chao [25] introduced a sleep posture recognition system based on an air spring mattress hardness adjustment mechanism that employed an embedded system for data collection and analysis. By defining supine and lateral positions and generating posture images using relative air pressure change rates followed by filtering, posture recognition was conducted using a Convolutional Neural Network (CNN), yielding an F1 score of 0.981. Hu [26] proposed a real-time, low-cost sleep posture recognition system based on pressure-sensitive conductive sheets and transfer learning. The system featured a cost-effective pressure sensor array made of conductive fabric and wires, arranged as a bedsheet with 1024 nodes. Data acquisition was facilitated by an Arduino Nano via a 10-bit analog-to-digital converter at a sampling rate of 0.4 frames per second. Employing a shallow CNN deployed on a personal computer, the system recognized six health-related sleeping postures for five participants. Traditional training–testing methods yielded an accuracy of 84.80%, which was enhanced to 91.24% through subject-specific transfer learning. Data collection was conducted through textile pressure sensors embedded within the sheet. The experimental setup included a sheet, 2.5 mm thick, hosting 64 × 27 textile-based pressure sensors that measured pressures ranging from 0 to 2 PSI and transmitted data via Wi-Fi to a computer system for processing. An Artificial Neural Network (ANN) model was used for posture classification, with nested cross-validation ensuring the model’s generalization capability. This model achieved a prediction accuracy of 97.9% on the test set, accompanied by a Cohen’s kappa coefficient of 97.2%. Matar [27] leveraged sheet-embedded pressure sensors for bed posture classification; however, commonly used sleep posture recognition methodologies rely heavily on extensive sensor networks for data acquisition, escalating system complexity and maintenance requirements. The dependency on dense sensor arrays escalates system complexity and maintenance requirements as each sensor necessitates proper calibration and upkeep to guarantee data precision, potentially leading to supplementary operational expenses over extended periods. Furthermore, approaches involving converting pressure data into images for model input can slow down the training process taken by Hu [26] and Matar [27]. The need for such data preprocessing and transformation may also impede the real-time responsiveness of sleep posture recognition systems.

**Figure 1:**
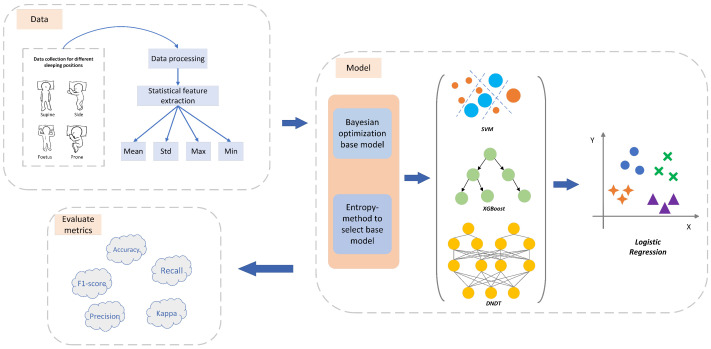
Developed architecture for enhanced stacked sleep recognition models.

Current methods for recognizing sleeping postures, while accurate, heavily rely on large sensor arrays, complicating system architecture and increasing maintenance challenges. Additionally, the requirement to convert pressure data into images hinders the real-time response. Addressing these issues, this study introduces a novel sleeping posture recognition model that uses just four airbag sensors, significantly reducing sensor dependence. We employ an entropy weighting method to select optimal base models and logistic regression for meta-learning to construct an enhanced stacking model. Experimental results demonstrate that our model substantially outperforms traditional single base models in accuracy and maintains high performance with fewer sensors. The overall architecture of the model designed in this research is shown in Figure 1.

## 3. Hardware Materials and Datasets

In this section, we will provide a detailed description of the equipment utilized for the experiment and the process of data acquisition.

### Experimental Material

**Figure 2:**
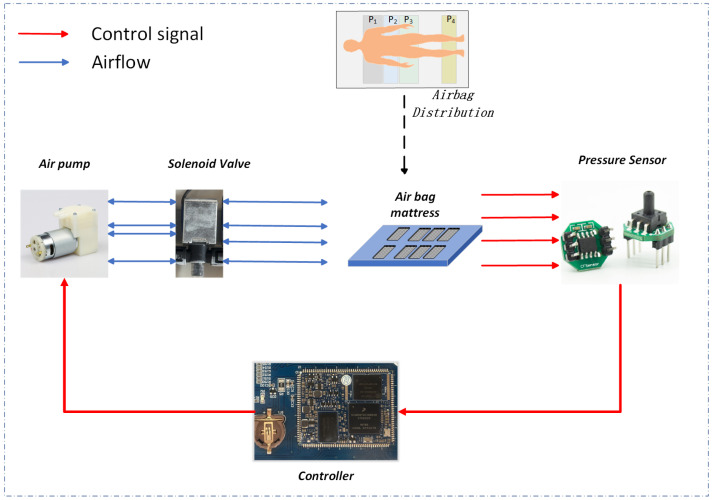
Airbag Mattress Workflow.

Figure 2 illustrates the connection diagram of the airbag mattress, including the solenoid valve, pressure sensor, air pump, and controller. The red arrow indicates the direction of airflow, while the blue arrow indicates the direction of the control signal. In this investigation, adhering to the principles of ergonomics and sleep comfort standards, we systematically gathered pressure distribution data over a duration of 20 s from 41 healthy young participants, comprising 25 males and 16 females, across four quintessential sleeping postures—Supine, Side, Fetus, and Prone.Each side of the mattress is equipped with four independently inflatable and deflatable airbag units. The focus of this study is on the four airbags on one side ($P_{1}$, $P_{2}$, $P_{3}$, and $P_{4}$), which correspond to the back, waist, buttocks and legs of the human body (as shown in the Airbag Distribution section of the figure). The sensors monitor the pressure changes of the airbags in real time and convert the data into electrical signals to be transmitted to the controller. Based on the received pressure data, the controller adjusts the air pump and solenoid valve to control inflation and deflation of the airbags to ensure that their pressure reaches initial set values required for experimentation. Taking inflation as an example, the workflow of the entire system is as follows:

1. The controller sends a control signal to activate the air pump and the corresponding solenoid valve based on the predetermined target pressure.
2. Activated by the control signal, the air pump generates airflow that is directed into the airbag via the open solenoid valve.
3. The pressure sensor continuously monitors the pressure inside the airbag and provides real-time data to the controller. This allows for accurate and immediate feedback on the pressure values within the airbag.
4. The controller receives real-time pressure data from the pressure sensor and then determines whether to continue inflating or if the target pressure value has been reached based on the real-time pressure data.
5. Once the target pressure is achieved, the controller issues a signal to stop the air pump and close the solenoid valve, stabilizing the airbag’s pressure.

## 4. Methodology

This section describes the principles of the enhanced stacked integration model, and individual base models.

### 4.1. Enhanced Stacked Integration Model

**Figure 3:**
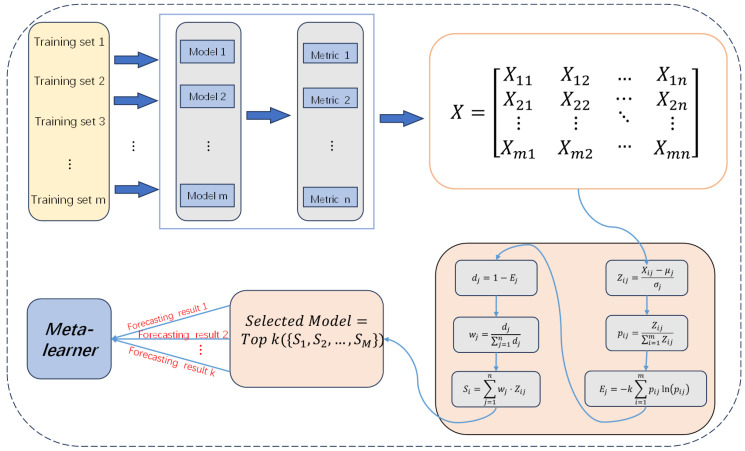
Stacking ensemble model structure.

Combining multiple learners is a commonly used strategy to enhance model performance, and it has been widely validated across various machine learning applications [28,29]. Stacked integrated modeling is an effective technique for improving model accuracy by incorporating the predictive power of multiple base learners. However, the choice of base model is crucial for the performance of stacked integrated models [30,31]. In this study, we utilize the entropy-weighted composite score [32] to select the base models. As shown in Figure 3, the models are trained from Model 1 to *Model m* using corresponding training sets from *Training set 1* to *Training set m*. The models are then trained using a series of metrics called Metrics and evaluated by a series of metrics *Metric 1* to *Metric n*. The results are aggregated into a matrix *X*. To eliminate the effects of different magnitudes and scales, normalization is applied to generate a $Z_{ij}$ matrix from the performance data. Subsequently, normalized weights $P_{ij}$ are computed for each normalized score and used to calculate the entropy $E_{j}$ for each metric, determining their importance. Based on these metrics’ importance, a weighted composite score $S_{i}$ is calculated for each model. According to the results of performance verification and empirical heuristic methods, this study ultimately selected a k value of 3. Subsequently, the three models with the highest comprehensive scores were identified as base models (specific values are presented in Table 1), which include XGBoost, SVM, and DNDT. After selecting the base models, their prediction results on training data are used as new features to generate a new training set [33]. The second layer uses these new features and original target variables to train a meta-learner that synthesizes outputs of base learners for final prediction. In the testing phase, test data pass through each base learner to generate predictions, which are then fed into the trained meta-learner to obtain final predictions. In this way, stacking modeling effectively utilizes the advantages of different base learners to improve the overall prediction accuracy.

**Figure 4:**
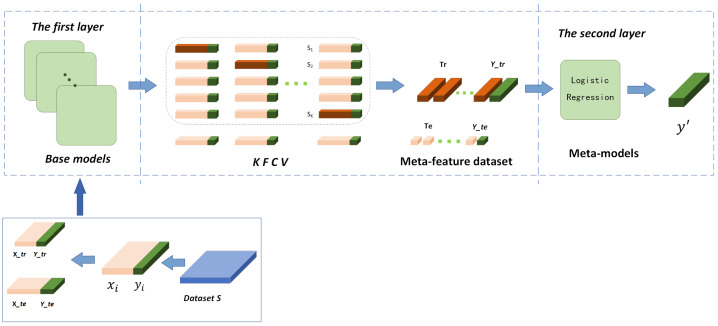
k-fold cross-validation training process.

To reduce the risk of overfitting due to data redundancy in the two-layer learning process, it is crucial to utilize the k-fold cross-validation technique for data segmentation and model training within the training phase of the stacking ensemble model [34]. As shown in Figure 4, given a complete dataset, $S={\left(y_{i},x_{i}\right)}_{i=1}^{N}$, where $y_{i}$ is the target value and $x_{i}$ is the feature. Divide the dataset S into the training set $\left(X_{tr},Y_{tr}\right)$ and testing set $\left(X_{te},Y_{te}\right)$. Then, the training set $\left(X_{tr},Y_{tr}\right)$ is divided into *K* folds, and each fold of the dataset is denoted as ${\left(X_{tr}^{k},Y_{tr}^{k}\right)}_{k=1}^{K}$. Typically, the number of K is chosen as either 5 or 10 for cross-validation purposes. Due to considerations of expense, this study implements the 5-fold cross-validation approach to train the stacking ensemble model. For each base model $B_{j}$ (there are n base models in total), the following operation is performed on the *k*-th fold: use all of the fold data except the *k*-th fold to train the base model $B_{j}$, $B_{j}^{k}=B_{j}{\left(\left(X_{tr}^{m},Y_{tr}^{m}\right)\right)}_{m\neq k}$, and then use the trained base model $B_{j}^{k}$ to predict the data of the *k*-th fold $X_{tr}^{k}$. Then, we obtain the prediction result matrix $P_{j,k}=B_{j}^{k}\left(X_{tr}^{k}\right)$, and splice the prediction results of all the folds together to obtain the prediction result matrix ${Tr}_{j}$ of the base model $B_{j}$ for the whole training set, and then merge all the data generated by the base models $B_{1},B_{2},\ldots B_{n}$ to obtain the new second layer training data *Tr* as follows:

(1) $${Tr}_{j}=\left[\begin{array}{c} P_{j,1}\\ P_{j,2}\\ \begin{array}{c} \vdots \\ P_{j,K} \end{array} \end{array}\right]$$

(2) $$Tr=\left[\begin{array}{cccc} {Tr}_{1,1} & {Tr}_{2,1} & \ldots & {Tr}_{n,1}\\ {Tr}_{1,2} & {Tr}_{2,2} & \ldots & {Tr}_{n,2}\\ \vdots & \vdots & \ddots & \vdots \\ {Tr}_{1,K} & {Tr}_{2,K} & \ldots & {Tr}_{n,K} \end{array}\right]$$

For each base model $B_{j}$, use the whole training set $\left(X_{tr},Y_{tr}\right)$ to train the base model $B_{j}$ and predict the test set $X_{te}$ to obtain the prediction result matrix ${Te}_{j}$. Next, combine the prediction results of all the base models on the test set to obtain the new second layer test data matrix *Te* as follows:

(3) $$Te=\left[\begin{array}{cccc} {Te}_{1,1} & {Te}_{2,1} & \ldots & {Te}_{n,1}\\ {Te}_{1,2} & {Te}_{2,2} & \ldots & {Te}_{n,2}\\ \vdots & \vdots & \ddots & \vdots \\ {Te}_{1,K} & {Te}_{2,K} & \ldots & {Te}_{n,K} \end{array}\right]$$

The meta-model *M* is trained using the combined training data *Tr* and predicted using the combined test data *Te* to obtain the final prediction $y^{\prime }$:

(4) $$\left[\begin{array}{cc} Tr & Y_{tr} \end{array}\right]\overset{train}{\to }M\overset{predict}{\to }\left[\begin{array}{c} Te \end{array}\right]\to y^{\prime }$$

### 4.2. Base Models

#### 4.2.1. DNDT

The DNDT (deep neural decision tree) model [35] merges the advantages of neural networks and decision trees, targeting both the extensive modeling power of neural networks and the inherent interpretability of decision trees for tabular data. DNDT maps the structure of a decision tree into a neural network, which is trained using gradient descent to maintain decision tree interpretability. This approach replaces traditional decision trees’ greedy splitting with a neural network that optimizes all parameters, enhancing the model’s effectiveness and clarity. The main steps are as follows:

1. Hard binning is not trivial, so a trivial approximation function is used. Given a continuous variable *x*, it is desired to partition it into *n* + 1 intervals using *n* split points $\beta_{1},\beta_{2},\ldots \beta_{n}$, which are trainable variables and satisfy monotonically increasing, i.e., $\beta_{1}<\beta_{2}<\ldots \beta_{n}$. A single-layer neural network is then constructed, using softmax as the activation function:
  (5) $$\pi =f_{w,b,\tau }\left(x\right)=softmax\left(\frac{wx+b}{\tau }\right)$$
  where *w* is a constant vector taking the values w=[1,2,…,n+1] and *b* is constructed as follows:
  (6) $$b=\left[0,-\beta_{1},-\left(\beta_{1}+\beta_{2}\right),\ldots ,-\left(\beta_{1}+\beta_{2}+\ldots +\beta_{n}\right)\right]$$
  τ is a temperature factor, and as τ→0, the output tends to a solo heat vector.
2. Construct a decision tree using the Kronecker product. Suppose there is an input instance $x\in R^{D}$ with D features. For each feature $x_{d}$, use its own neural network $f_{d}\left(x_{d}\right)$ for binning:
  (7) $$z=f_{1}\left(x_{1}\right)⊗f_{2}\left(x_{2}\right)⊗\cdots ⊗f_{D}\left(x_{D}\right)$$
  Here, *z* is also an approximate unique heat vector indicating the index of the leaf node at which instance *x* arrives. Finally, each leaf node *z* is assumed to have a linear classifier classifying instances arriving at that leaf node.
3. The parameters of the segmentation point and leaf node classifiers are learned simultaneously through backpropagation. Since all forward-passing steps are microscopic, all parameters can be trained using stochastic gradient descent (SGD). This allows the DNDT model to achieve decision tree interpretability while leveraging the training of neural networks to enhance the overall performance of the model.

In this paper, we aim to optimize the parameters (num_trees, tree_depth, learning_rate, batch_side) of the DNDT model in order to ensure efficient and stable convergence within a reasonable timeframe. Our goal is to prevent over- or underfitting and achieve optimal performance on a given task.

#### 4.2.2. XGBoost

XGBoost is an iterative integration algorithm that enhances the robustness of tree-based integration algorithms by combining multiple weak learners [36]. This algorithm effectively maximizes the objective function concurrently by utilizing both Gradient Boosting Decision Tree (GBDT) and regularization strategies. XGBoost’s primary advantage is its capacity to streamline the learning model and mitigate overfitting through the integration of diverse regularization elements.

The fundamental idea of XGBoost is to progressively incorporate trees and iteratively refine features to construct subsequent trees. This process entails adjusting to the residuals left by the preceding tree and then deriving the ultimate prediction score by combining the outputs from several trees. The XGBoost model can be represented by the following equation:

(8) $${\hat{y}}_{i}^{\left(t\right)}=\sum_{k=1}^{K}f_{k}\left(x_{i}\right)={\hat{y}}_{i}^{\left(t-1\right)}+f_{t}\left(x_{i}\right)$$

where *K* represents the number of trees, $f_{k}\left(x_{i}\right)$ and $f_{t}\left(x_{i}\right)$ denote the predicted values of the *k*-th and *t*-th trees on the *i*-th sample, and $x_{i}$ denotes the input variable. ${\hat{y}}_{i}^{\left(t\right)}$ and ${\hat{y}}_{i}^{\left(t-1\right)}$ represent the predicted values of the *i*-th sample at the *t*-th and (*t* − 1)-th iterations, respectively.

The objective function of the XGBoost model is expressed as the following equation:

(9) $${Obj}^{\left(t\right)}=\sum_{i-1}^{n}l\left(y_{i},{\hat{y}}_{i}^{\left(t\right)}\right)+\sum_{k-1}^{K}\Omega \left(f_{k}\right)$$

where ${Obj}^{\left(t\right)}$ is the objective function and $\sum_{i-1}^{n}l\left(y_{i},{\hat{y}}_{i}^{\left(t\right)}\right)$ is the loss function, which is used to measure the gap between predicted and actual values, and to optimize the fit of the weak learner.

The regularization term $\Omega (f_{k})$ is represented as:

(10) $$\Omega \left(f\right)=\delta T+\frac{1}{2}\lambda \sum_{j=1}^{T}\omega_{j}^{2}$$

In this context, Ω symbolizes the regularization term. T indicates the total count of leaf nodes, and ω represents the weight assigned to these nodes. Moreover, δ and λ act as regularization parameters, tasked with managing the complexity related to both the number of leaf nodes and the weights of these nodes. This is carried out to enhance computational efficiency and improve the model’s generalization ability. In this paper, we focus on optimizing XGBoost parameters such as *learning*_*rate*, max_depth, and n_estimators.

#### 4.2.3. SVM

The support vector machine (SVM) is a widely used supervised learning algorithm for classification and regression tasks [37]. Its basic principle is to maximize the spacing of classification boundaries by identifying an optimal hyperplane to classify the data. SVM can be applied to both linearly and nonlinearly separable datasets, achieving this by mapping the data to a high-dimensional space using a kernel function trick that makes it linearly separable in that space. In situations where differentiation is nonlinear, SVM employs a kernel function $K(x_{i},x_{j})$ to project the data into a higher-dimensional space. Widely utilized kernel functions encompass linear, polynomial, and radial basis function (RBF) kernels. Consequently, the optimization challenge then becomes:

(11) $$min\frac{1}{2}{∥\omega ∥}^{2}+C\sum_{i-1}^{n}\xi_{i}$$

Subject to the constraints being satisfied:

(12) $$y_{i}\left(\omega \cdot \phi \left(x_{i}\right)+b\right)\geq 1-\xi_{i}\;,\forall_{i}$$

Here, $\phi \left(x_{i}\right)$ is the kernel function mapping, $\xi_{i}$ is the slack variable, and *C* is the penalty parameter to balance the effects of interval maximization and misclassification errors. This paper focuses on optimizing SVM parameters including *C*, kernel, and γ.

## 5. Case Study

This section provides a description of the datasets utilized, the models compared, the base model for classification, the model parameter settings, and the evaluation metrics. Additionally, this experiment was conducted on a Windows system using Pycharm as the programming environment, with 16 Gigabytes of RAM and a 3.2 GHz AMD Ryzen 7 5800H with Radeon Graphics CPU.

### 5.1. Dataset Description

**Figure 5:**
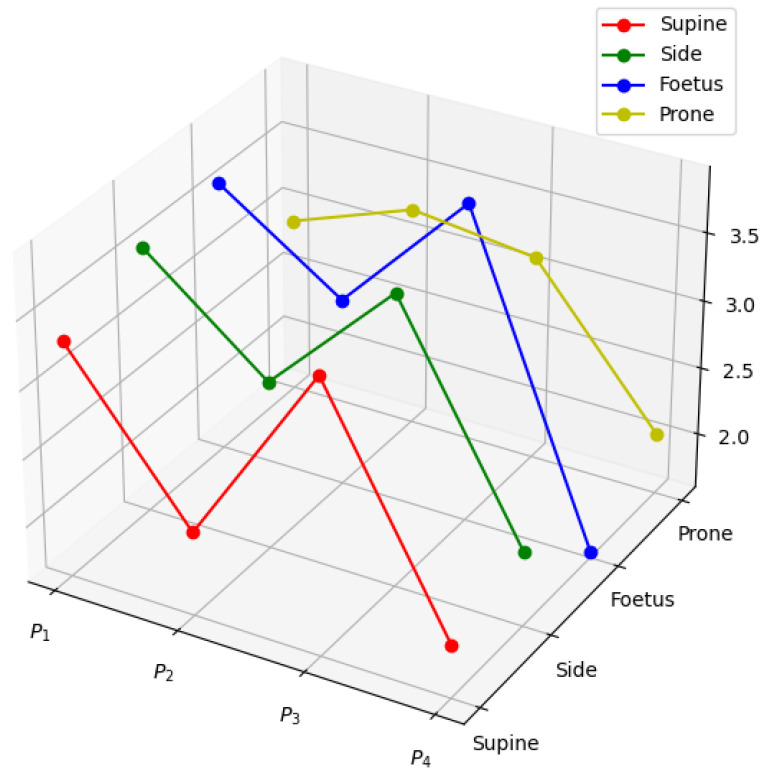
Air pressure distribution in different sleeping positions.

In the process of data collection, we initially set the air pressure at (1 ± 0.1) kPa. A random selection of data from a subject was visualized to create Figure 5 showing the pressure distribution of four air sacs ($P_{1}$, $P_{2}$, $P_{3}$, and $P_{4}$) in supine, lateral, fetal, and prone positions. The results indicated that the supine position exhibited higher pressure in the $P_{1}$ and $P_{3}$ air sacs, with pressures of 3.32 kPa and 3.61 kPa, respectively. Additionally, the maximum pressure value of the $P_{2}$ air sac was 3.22 kPa in the prone position, while the minimum pressure value of the $P_{4}$ air sac was 1.64 kPa in the fetal position.

The function of feature extraction in machine learning models is to enrich the dataset by expanding the quantity and diversity of input features, thus improving the model’s predictive accuracy and its ability to generalize. The authors of [38,39,40] reported utilizing a set of statistical features such as mean, standard deviation, minimum, and maximum sensor values. They found that using statistical features resulted in high accuracy, particularly when applied to a limited number of sensors. Consequently, we also rely on a simple set of statistical features for our classification. The original dataset *D* contains pressure values from four airbags in various sleeping positions, denoted as:

(13) $$D=\left\{P_{ij}|i=1,2,\cdots ,n;j=1,2,3,4\right\}$$

where $P_{ij}$ denotes the pressure value of the *j*-th airbag in the *i*-th sample and *n* is the total number of samples.

Statistical attributes are vital in encapsulating key insights about the data, which assists the model in acquiring a deeper comprehension of the data’s distribution and traits. This ultimately leads to an improvement in prediction performance. Specifically, we extracted the maximum ($P_{max}$), minimum ($P_{min}$), mean ($P_{mean}$), and standard deviation ($P_{std}$) of the four airbags in each sleeping position, which are expressed as follows:

(14) $$P_{max,i}=\max\left(P_{i1},P_{i2},P_{i3},P_{i4}\right)$$

(15) $$P_{min,i}=\min\left(P_{i1},P_{i2},P_{i3},P_{i4}\right)$$

(16) $$P_{mean,i}=\frac{1}{4}\sum_{j=1}^{4}P_{ij}$$

(17) $$P_{std,i}=\sqrt{\frac{1}{4}\sum_{j=1}^{4}{\left(P_{ij}-P_{mean,i}\right)}^{2}}$$

The final constructed feature set $D_{new}$ contains the original data and its extracted features with a total of eight features, which are denoted as:

(18) $$D_{new}=\left\{\left[P_{i1},P_{i2},P_{i3},P_{i4},P_{max,i},P_{min,i},P_{mean,i},P_{std,i}\right]|i=1,2,\cdots ,n\right\}$$

By performing feature extraction, we are able to transform the original dataset D into a new feature set $D_{new}$ that contains additional information. This enhancement ultimately contributes to the improved performance of the classification model.

### 5.2. Model Comparison

In this study, we employed the developed enhanced stacking model to evaluate it against the base model using five different metrics. The purpose was to demonstrate that the enhanced stacking model outperforms the base model in sleep recognition across these metrics. Additionally, we conducted comparisons with other integrated algorithms (LightGBM, AdaBoost, Bagging, Hard Voting, Soft Voting).

### 5.3. Base Model for Classification

In this study, a total of seven models were selected as candidates for the base model: Extreme Gradient Boost (XGBoost), Random Forest (RF), support vector machine (SVM), Extreme Learning Machine (ELM), Multi-Layer Perceptron (MLP), deep neural decision tree (DNDT), and a Deep Learning Model with Adaptive Learning Mechanisms (Tabnet). The performances of each candidate base model were evaluated using collected datasets in different sleeping positions. The entropy weighting method was then applied to weight the five performance metrics of each model, resulting in an entropy-weighted composite score for each base learner. Based on the entropy-weighted composite score, the base learner with the best performance was selected as the first layer base model of the proposed enhanced stacking model. Multi-categorical logistic regression was chosen as the meta-learner due to its advantages of being a simple model, efficient training, resistance to overfitting, strong stability, and good interpretation. A regularization strength C value of 10 and L2 regularization type were used for the meta-learner.

### 5.4. Model Parameter Optimization

Hyperparameter settings are designed to enhance the model’s precision by pinpointing the most advantageous settings for each parameter. Therefore, when configured with appropriate hyperparameters, the model is capable of accurately calculating optimal weights using the provided training data and algorithms. Usually, hyperparameter values are determined using either empirical knowledge or iterative experimentation. Both approaches are labor-intensive, and manually determining the optimal hyperparameter values can require extensive time. However, these difficulties are being mitigated by recent advancements in optimization techniques [41]. In recent years, Bayesian optimization (BO) has become increasingly popular for hyperparameter tuning in machine learning models. This global optimization technique aims to minimize the unknown function h(x).

(19) $$x^{*}={argmin}_{x\in \chi }\;h\left(x\right)$$

where $x^{*}$ denotes the input *x* for which h(x) makes a minimum, x∈χ means that *x* is in the feasible domain χ, and $h\left(x\right)$ is the objective function, which is the function we wish to minimize. In this paper, *Bo* is the parameter being used to optimize the candidate model. Specifically, the names of the parameters and their corresponding optimal values are in Table 2. The number of optimization iterations for each model is set to 200, ensuring adequate tuning for performance improvement.

### 5.5. Parameters of Candidate Models

In this paper, the recognition of sleeping positions is approached as a multiclassification problem, where each sleeping position represents a category. The effectiveness of the model is assessed using five indicators: accuracy, precision, F1-score, recall, and kappa coefficient [38]. The equations for these metrics are detailed below:

(20) $$Accuracy=\;\frac{TP+TN}{TP+FP+TN+FN}$$

(21) $$Precision=\frac{TP}{TP+FP}$$

(22) $$F1\text{-}score=\frac{2*TP}{2*TP+FP+FN}$$

(23) $$Recall=\frac{TP}{TP+FN}$$

(24) $$kappa=\frac{P_{o}-P_{e}}{1-P_{e}}$$

$P_{o}$ represents the observed consistency ratio, indicating accuracy, while $P_{e}$ denotes the consistency ratio derived from random chance expectation. This is calculated based on the edge probabilities for each category. The kappa coefficient not only evaluates the model’s consistency across different prediction categories but also considers chance consistency, thus providing a more rigorous and reliable assessment of performance.

## 6. Results and Discussion

To validate the performance of the proposed stacked model in recognizing sleeping positions, this experiment was designed with four parts: (i) selection of the base model based on the entropy weighting method, (ii) analysis of the confusion matrix between the augmented stacked model and the first layer of the base model for classification of sleeping positions, (iii) comparison of the performance of the seven candidate models and the enhanced stacking model as well as comparison with other integrated algorithms, and (iv) plotting Sankey diagrams to show the contribution of features to the base model.

### 6.1. Base Model Selection

**Figure 6:**
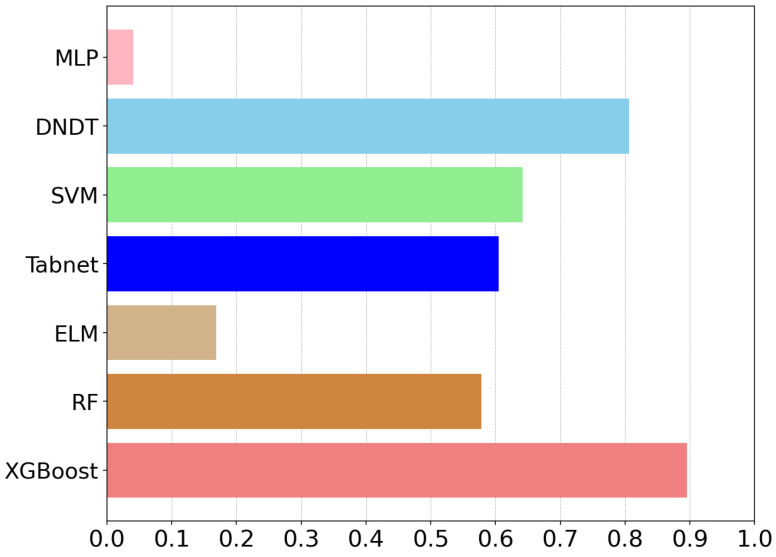
Entropy-weighted composite scores for different models.

We randomly divided the dataset $D_{new}$ into a training set and a test set in the ratio of 8:2. The effectiveness of the learners from the seven candidate bases was evaluated using five criteria: accuracy, precision, recall, F1-score, and kappa coefficient. Each algorithm underwent 10 iterations, and the mean values of the five metrics were calculated. We utilized the entropy weighting technique to impartially determine the weights for the five indicators of each model. The weighting coefficients were calculated based on the internal variables of the five distinct indicators, specifically the information entropy associated with each indicator. The entropy-weighted aggregate score was derived by multiplying each performance metric by its respective weighting factor. The specific scores of each model are shown in Table 1 and Figure 6. From these weighted composite scores, it is evident that XGBoost had the best composite performance with a score of 0.8955, followed by DNDT and SVM with scores of 0.8060 and 0.6418, respectively. Therefore, for our sleep recognition model, we have chosen XGBoost, SVM, and DNDT as our base models based on their superior performance in terms of weighted composite scores.

### 6.2. Analysis of Sleeping Position Classification Results

**Figure 7:**
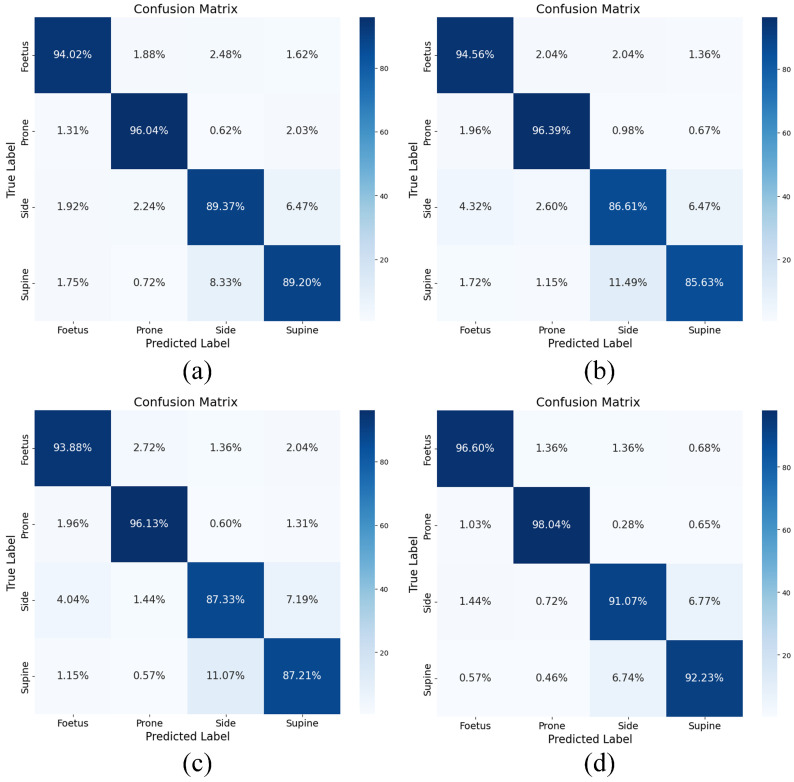
Confusion matrix for: (**a**) XGBoost. (**b**) SVM. (**c**) DNDT. (**d**) Stacking.

We conducted a comparison of the performance between the proposed enhanced stacking model and its first layer of base models in various sleeping position classification tasks using the confusion matrix. As shown in Figure 7d, the enhanced stacking model demonstrated significantly superior performance compared to the other base models in classifying different sleeping postures. Specifically, the accuracies of the model for the fetus pose, prone pose, side pose, and supine pose were 96.60%, 98.04%, 91.07%, and 92.23%, respectively.

In terms of misclassification, it was observed that the XGBoost and DNDT models exhibited more errors when classifying side and supine poses, with side poses being misclassified as supine poses and vice versa for supine poses being misclassified as side poses. The SVM model exhibited a particularly high rate of misclassification for the supine pose, with 11.49% of the supine pose samples being misclassified as side poses. In contrast, the enhanced stacking model demonstrated significantly fewer misclassifications, especially with the fewest misclassifications of the fetus and prone poses. Furthermore, it showed higher classification accuracy for supine and side poses compared to the other three base models. Specifically, in the enhanced stacking model, 6.77% of the side pose samples were misclassified as supine poses and 1.44% were misclassified as fetus poses; additionally, 6.74% of the supine pose samples were misclassified as side poses. By leveraging the strengths of multiple base models, the enhanced stacking model is capable of effectively handling complex and diverse features while enhancing classification accuracy and robustness.

### 6.3. Model Comparison

**Figure 8:**
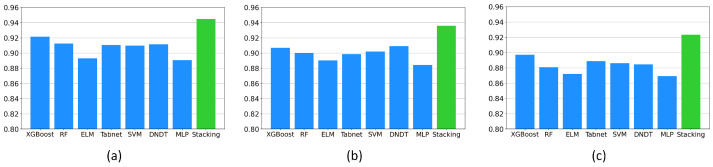
(**a**) Compares the accuracy of different models. (**b**) Compares the F1 scores of different models. (**c**) Compares the kappa coefficients of different models. The optimal values are indicated in green color.

To further assess the proposed enhanced stacked integration model, we selected accuracy, precision, recall, F1-score, and kappa coefficients to evaluate the performance of each model. Figure 8 illustrates the performance of each model on accuracy, F1-score, and kappa coefficients, with green indicating the best performing model. The specific values of the five metrics for the eight models are presented in Table 3. The augmented stacking model achieves an accuracy of 0.9448, a precision of 0.9406, a recall of 0.9319, an F1-score of 0.9362, and a kappa coefficient of 0.9235.

These results demonstrate that the augmented stacking model outperforms other models in terms of overall prediction correctness on the sleeping position recognition task as well as positive class prediction accuracy and coverage, consistency, and reliability, among other aspects. This superiority can be attributed to the fact that the augmented stacking model addresses bias and variance problems by combining predictions from multiple base models while utilizing diverse models to comprehensively capture complex patterns in data.

**Figure 9:**
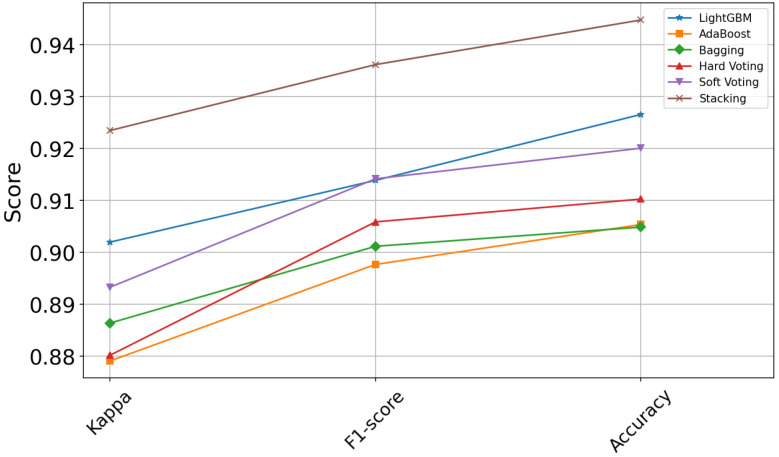
Enhanced stacking model vs. other integrated models.

In addition to XGBoost and RF in the candidate models, we also compare with other integrated models, mainly Bagging Decision Tree (TreeBag), Adaptive Augmented Classifier (AdaBoost), LightGBM, and Voting (Hard Voting, Soft Voting). We still use three metrics: F1-score, kappa, and accuracy for model comparison. As shown in Figure 9, this proposed enhanced stacking model outperforms other integrated models in terms of performance. From Table 4, it is concluded that the enhancement ranges of the enhanced stacking model in accuracy, F1-score, and kappa with respect to the other integrated models are between 1.82% and 3.99%, 2.2% and 3.85%, and 2.15% and 4.44%, respectively. The enhancement of the kappa value is more obvious; the kappa value of the enhanced stacking model is 0.9235, which has a greater advantage compared to other models such as LightGBM (0.9020) and Soft Voting (0.8933). These results indicate that the enhanced stacking model has some advantages in consistency and overall performance, making it a preferred model for dealing with sleeping position recognition tasks.

### 6.4. Characteristic Importance Analysis

In order to gain a more comprehensive understanding of the performance of the augmented stacking model in the task of recognizing sleeping positions, we conducted an analysis on the importance of features. Firstly, we utilized the built-in feature importance property of the XGBoost model to calculate the contribution of features to decision tree splitting during model training. For the SVM and DNDT models, we employed the Permutation Importance method to compute feature importance by randomly replacing specific feature values and observing their effect on model performance. Subsequently, we normalized the feature importance of each base model and calculated the average importance of each feature among all base models. The results are presented in Table 5.

**Figure 10:**
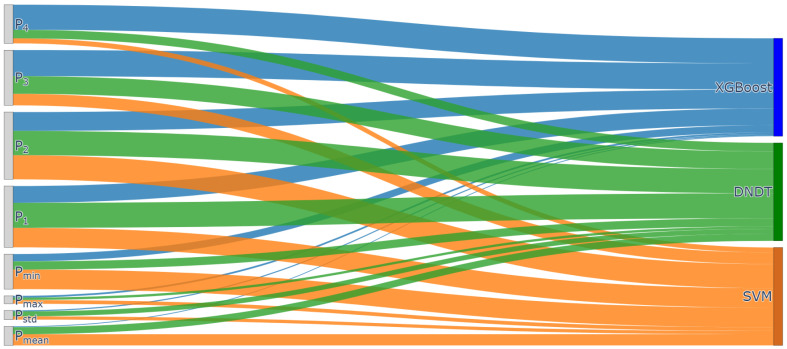
Contribution of features to the first base model.

To further visualize the contribution of features in different base models, Sankey’s Figure 10 was plotted to illustrate how features contribute to each base model (XGBoost, SVM, and DNDT). The importance value of each feature is depicted by the streamlines in the figure, with the width of the streamlines representing the magnitude of importance for each feature. In the first layer of the base model of the enhanced stacking model, different features contribute variably to each base model, as shown in Table 5; when utilizing the XGBoost, SVM, and DNDT models for classifying sleeping postures, it was observed that features $P_{3}$, $P_{2}$, and $P_{1}$ made the most significant contributions to the models, with respective contributions of 0.2671, 0.2463, and 0.2556. These features correspond to the hips, lumbar, and back regions of the human body, respectively. This finding emphasizes that different body parts have varying contributions in different models. Furthermore, the statistical feature ($P_{min}$) plays a crucial role in all models with an average contribution of 0.1194, indicating that statistical features are essential for improving model performance.

## 7. Conclusions and Future Work

This paper introduces a novel model for sleep posture recognition, specifically designed for use with an airbag mattress. The proposed model utilizes the entropy weighting method to assess the performance of multiple base learners and subsequently selects the top three to construct a stacking ensemble. Experimental results indicate that the model achieves accuracy, F1-score, and Kappa coefficient values of 94.48%, 93.62%, and 92.35%, respectively. This model has been successfully integrated into a smart mattress system, which is now commercially available. The smart mattress incorporates a sensor fault self-check module that continuously monitors the operational status of its sensors in real time. In cases where a sensor failure occurs—such as data anomalies or lack of output—the system automatically compensates for pressure data while issuing an alert, thereby ensuring that overall functionality remains unaffected by individual sensor faults. This design markedly improves both the reliability and robustness of the system.

Although the enhanced stacking ensemble learning model exhibits robust performance in sleep posture classification, it is not without potential limitations related to sensor malfunctions, air mattress leakage, and data loss. These challenges may result in diminished model efficacy, inaccurate monitoring outcomes, and decreased patient comfort. Consequently, future research will prioritize addressing these shortcomings with the aim of enhancing the model’s stability and predictive accuracy.

## Figures and Tables

**Table 1:** Entropy-weighted composite scores of candidate base learners.

Model	Scores
Xgboost	0.8955
RF	0.5783
ELM	0.1691
Tabnet	0.6050
SVM	0.6418
DNDT	0.8060
MLP	0.0403

**Table 2:** Parameters of candidate models.

ML Algorithms	Hyperparameters	Scope of Values	Optimal Values
XGBoost	learning_rate max_depth n_estimators	(0.001, 0.5) (1, 10) (50, 500)	0.169 5 300
SVM	C Gamma kernel	(0.1, 10) (0.001, 1) (linear, poly, rbf)	4.729 0.467 rbf
RF	max_depth min_samples_leaf min_samples_split n_estimators	(1, 20) (1, 5) (2, 10) (50, 300)	7 3 5 269
ELM	activation_func n_neurons	(100, 500) (sigm, tanh)	215 tanh
MLP	activation alpha solver	(relu, tanh) (0.0001, 0.01) (adam, sgd)	Relu 0.0084 adam
Tabnet	dim_num_decision_steps dim_relaxation_factor dim_sparsity_coefficient	(3, 15) (1, 5) (1 × 10^−5^, 1 × 10^−1^)	9 1 1.256 × 10^−5^
DNDT	num_trees tree_depth learning_rate batch_size	(1, 10) (1, 10) (0.0001, 0.01) (16, 32, 64, 128)	7 1 0.0025 16

**Table 3:** Specific indicator values for different models.

Model	Accuracy	Precision	Recall	F1-Score	Kappa
XGBoost	0.9215	0.9139	0.9102	0.9070	0.8972
RF	0.9126	0.9083	0.8926	0.9003	0.8810
ELM	0.8931	0.8849	0.8961	0.8904	0.8721
Tabnet	0.9108	0.8920	0.9056	0.8987	0.8891
SVM	0.9079	0.8949	0.9097	0.9022	0.8862
DNDT	0.9115	0.9013	0.9171	0.9091	0.8846
MLP	0.8907	0.8724	0.8965	0.8843	0.8693
**Stacking (Ours)**	**0.9448**	**0.9406**	**0.9319**	**0.9362**	**0.9235**

**Table 4:** Specific indicator values for other integrated models.

Model	F1-Score	Kappa	Accuracy
LightGBM	0.9139	0.9020	0.9266
AdaBoost	0.8977	0.8791	0.9054
Bagging	0.9012	0.8864	0.9049
Hard Voting	0.9059	0.8802	0.9103
Soft Voting	0.9142	0.8933	0.9201
**Stacking (Ours)**	**0.9362**	**0.9235**	**0.9448**

**Table 5:** Characteristic contribution values.

Features	$P_{1}$	$P_{2}$	$P_{3}$	$P_{4}$	$P_{\mathrm{mean}}$	$P_{\max}$	$P_{\min}$	$P_{\mathrm{std}}$
XGBoost	0.1713	0.1924	**0.2671**	0.2567	0.0086	0.0186	0.0745	0.0107
SVM	0.1998	**0.2463**	0.1181	0.0511	0.1139	0.0374	0.1997	0.0336
DNDT	**0.2556**	0.2434	0.1791	0.0873	0.0718	0.0242	0.0839	0.0498
Average	0.2089	0.2274	0.1881	0.1317	0.0648	0.0267	0.1194	0.0314

## Data Availability

The data that support the findings of this study are available upon request from the corresponding authors. The data are not publicly available due to ongoing follow-up research.
